# Sustainable Processing Approaches in White Winemaking: Impact of Oak Aging and Ultrasound-Assisted Treatment on Phenolic Compounds

**DOI:** 10.3390/foods15101709

**Published:** 2026-05-13

**Authors:** Camelia Elena Luchian, Elena Cornelia Focea, Bettina-Cristina Buican, Laurian Vlase, Elena Cristina Scutarașu, Lucia Cintia Colibaba, Ana-Maria Vlase, Valeriu V. Cotea

**Affiliations:** 1 ”Ion Ionescu de la Brad” Iași University of Life Sciences, 3rd M. Sadoveanu Alley, 700490 Iași, Romania; camelia.luchian@iuls.ro (C.E.L.); tarcancornelia2@gmail.com (E.C.F.); bettina.buican@iuls.ro (B.-C.B.); cristina.scutarasu@iuls.ro (E.C.S.); cintia.colibaba@iuls.ro (L.C.C.); 2 Department of Pharmaceutical Technology and Biopharmaceutics, University of Medicine and Pharmacy, 8 Victor Babes Street, 400347 Cluj-Napoca, Romania; 3 Department of Pharmaceutical Botany, Faculty of Pharmacy, Iuliu Hatieganu University of Medicine and Pharmacy, 8 Victor Babes Street, 400347 Cluj-Napoca, Romania; gheldiu.ana@umfcluj.ro

**Keywords:** phenolic compounds, wine maturation, chips, winemaking optimization, ultrasound-assisted extraction

## Abstract

Sustainability challenges in the wine sector have intensified the need for alternatives to conventional oak barrel maturation, a practice associated with high wood consumption, long maturation periods, and considerable economic and environmental cost. This study evaluates a resource-efficient maturation strategy for white wine using an experimental design comparing conventional oak alternatives with ultrasound-assisted extraction. Experiments were conducted in triplicate (*n* = 3) considering oak type (French chips vs. granules), dosage, toasting level (fresh, light, medium), and contact time (10 vs. 20 days). To enhance mass transfer, a 15 min ultrasound treatment (35 kHz) was applied. Statistical analysis (ANOVA One Way) indicated that oak fragment type and contact time significantly governed phenolic extraction (*p* < 0.05). Gallic acid concentrations increased significantly from 1.54 ± 0.03 mg L^−1^ in the control to 4.41 ± 0.12 mg L^−1^ in the most intensive ultrasound-assisted extraction treatment (*p* < 0.05). Syringaldehyde concentrations also showed a significant rise (1.13 to 1.44 mg L^−1^; *p* < 0.05). Ultrasound significantly accelerated extraction kinetics while mitigating the loss of flavan-3-ols (≤28%) compared to conventional oak treatments (up to 34%). Economic assessment demonstrated a substantial reduction in production costs, from 0.21–0.56 € L^−1^ range for standard fragment treatments to 0.05–0.07 € L^−1^ when ultrasound was applied. Cost-efficiency metrics (<0.03 € mg^−1^ gallic acid) confirmed that the combination of ultrasound and alternative oak materials provides an optimal, statistically significant balance between phenolic yield and economic viability.

## 1. Introduction

The continuous development of modern winemaking technologies is driven by the need to improve wine quality while reducing production time and costs. Traditional wine maturation is a time-intensive process that often conflicts with current market demands for efficiency and rapid product turnover. Consequently, there is increasing interest in innovative strategies capable of accelerating maturation processes without compromising sensory and chemical quality [1].

Among non-thermal technologies applied in winemaking, ultrasound has attracted considerable attention due to its simplicity of implementation and its ability to enhance process efficiency [2,3,4,5]. In particular, its use during maceration has been widely investigated in red wine production, where acoustic cavitation disrupts grape skin cell walls and vacuolar membranes. The resulting mechanical effects enhance the extraction of anthocyanins, tannins, and other intracellular phenolic compounds, reduce maceration time, and increase early-stage extraction yields [6,7,8]. However, when applied during oak maturation, ultrasound acts through a different mechanism, promoting solvent penetration into the wood matrix and facilitating the release of wood-derived compounds, including gallic acid, ellagitannins, and phenolic aldehydes [9,10]. Although previous studies have demonstrated the potential of ultrasound to accelerate extraction from plant and wood matrices, its effectiveness under specific enological conditions, as well as its impact on the phenolic profile and process efficiency, remains insufficiently characterized [4,11,12,13,14,15].

Phenolic compounds play a fundamental role in wine quality, influencing sensory attributes, oxidative stability, and aging potential. In white wines, although present at lower concentrations than in red wines, phenolics significantly contribute to oxidation reactions, browning, and aroma development through interactions with volatile compounds [16,17,18,19]. Moreover, oak maturation introduces additional phenolic constituents, which can further modify the wine’s chemical composition and sensory profile [16,20].

Iași viticultural region, situated in northeastern Romania on the Moldavian Plateau, exhibits a temperate continental climate with warm summers, cold winters, and moderate annual precipitation (500–600 mm), creating optimal conditions for cultivating aromatic acid-balanced white grape varieties [21]. Among these, Fetească regală is highly valued for its adaptability, moderate phenolic content, and capacity to produce wines with fresh acidity and delicate aromatic complexity [22]. These traits make it particularly suitable for experimental aging with alternative oak materials, such as chips and granular fragments, as its neutral yet expressive aroma profile allows precise evaluation of oak-derived phenolic compounds without masking the grape’s inherent sensory characteristics [23].

Based on these considerations, this study is guided by the hypothesis that ultrasound-assisted oak maturation enhances the extraction of phenolic compounds from alternative oak materials (chips and fragments), thereby reducing processing time while maintaining or improving wine quality. Furthermore, the novelty of this work lies in the combined evaluation of ultrasound treatment and alternative oak materials under controlled enological conditions, together with an integrated assessment of phenolic composition and process efficiency, aspects that have not been sufficiently addressed in previous studies.

The aim of this study was to evaluate the effect of ultrasound-assisted maturation using alternative oak materials (chips and fragments) on the phenolic profile of Fetească regală wine, while also assessing its potential to improve process efficiency and sustainability compared to conventional maturation techniques.

## 2. Materials and Methods

### 2.1. Wine Samples

**Figure 1:**
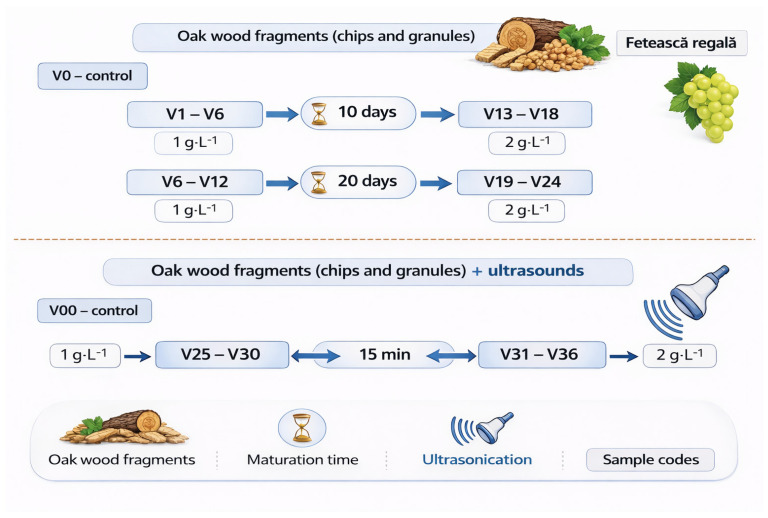
Schematic representation of Fetească regală wine maturation with oak wood fragments (chips and granules) with and without ultrasonication.

A total of 38 wine variants (Table 1), derived from the same base wine batch, were produced, including two untreated control samples (V0 and V00). All wines originated from Fetească regală grapes harvested at full technological maturity during the 2021 vintage. Each variant represented an independent treatment condition, and all analyses were performed in triplicate. The grapes had a sugar concentration of 240 g L^−1^ and met optimal sanitary conditions. The grapes originated from the Iași wine region, located in Vișan, Romania (47.0827° N, 27.6232° E), within the Moldavian Hills viticultural area. The experimental design consisted of a factorial combination of three factors: (i) oak fragment type (chips or granules), (ii) toasting level (fresh, light, medium), and (iii) dosage (1 or 2 g L^−1^). Two contact times (10 and 20 days) were evaluated under conventional maturation conditions. Additional treatments included ultrasound-assisted extraction (35 kHz, 15 min). In total, 36 treated variants and two controls were produced (Figure 1).

After harvest, the grapes were partially crushed and destemmed using a Delta E2 crusher–destemmer (Bucher Vaslin, Chalon-sur-Saône, France). The must was subjected to a pre-fermentative maceration for 5 h. The must was then separated from the solid fraction and clarified by gravity settling for 24 h at 10 °C. Alcoholic fermentation was carried out in stainless steel tanks. The must was inoculated with *Saccharomyces cerevisiae* yeast. The commercial strain Excellence^®^ B2 (Lamothe-Abiet^®^, Bordeaux, France) was added at a dose of 20 g h L^−1^, in accordance with the manufacturer’s recommendations and current legislation. Fermentation was conducted at a controlled temperature of 14 °C until dryness. After completion of alcoholic fermentation, the wine was racked off the coarse lees and allowed to stabilize. Sulfur dioxide (1 mL L^−1^, 6% solution) was then added to ensure microbial stability and oxidative protection. The wine was subsequently divided into 5 L glass vessels. Oak wood fragments, including chips and granules, were added at different doses (1 g L^−1^ and 2 g L^−1^). Three toasting levels were applied (light, fresh, and medium) (V1–V24). Two contact times were evaluated (10 and 20 days), as shown in Table 1. For comparison, additional samples (V25–V36) treated with the same types and doses of oak fragments were subsequently subjected to ultrasound treatment for 15 min. Ultrasonic treatment was performed using a Bandelin Sonorex RK 1028 C (Bandelin Electronic GmbH & Co. KG, Berlin, Germany) operating at 35 kHz and 1450 W thermal power (power density 16.7 W L^−1^, energy input 2.2 A). The bath (45 L nominal capacity) was filled with deionized water to a working volume of 30 L, which served as the acoustic transmission medium. Temperature was continuously monitored and maintained below 20 °C. Samples (5 L) were processed in sealed glass vessels, corresponding to a sample-to-bath volume ratio of 1:6. Vessels were placed in a perforated basket, positioned 1–2 cm above the tank bottom, and arranged centrally with at least 2–3 cm spacing from each other and the walls to ensure uniform cavitation. The immersion depth was adjusted so that the liquid level inside the vessels was equal to or slightly below the surrounding water level.

The oak fragments (Oeno Bois, Saint-Maurice, France) used in this study were derived from *Quercus robur* (French oak), a species widely recognized for its use in cooperage [24]. The wood was naturally matured outdoors for 24 months prior to processing. Two types of fragments were employed: chips and granules. The chips were produced in three toasting levels (fresh, light, and medium) and had sizes (5 × 5 × 1 mm—width × length × thickness). The granules measured between 3 and 8 mm and similarly facilitate the quick release of wood constituents during maturation. The wines were filtered, bottled, and stored at a temperature of 10 °C.

### 2.2. Physico-Chemical Parameters

Physico-chemical analyses were performed on all experimental variants in accordance with the methods described in the Compendium of International Methods of Analysis of Wines and Musts of the OIV. The parameters evaluated included pH, total and volatile acidity, alcoholic strength, residual sugars, total and free SO_2_, and phenolic compounds [25]. Alcohol content was measured by distillation followed by densimetric determination (OIV-MA-AS312-01). Volatile acidity was determined by steam distillation (OIV-MA-AS313-02), while titratable acidity (g tartaric acid L^−1^) was assessed by acid–base titration (OIV-MA-AS313-01). Residual sugars (g glucose L^−1^) were quantified using enzymatic assays (OIV-MA-AS311-01). The pH was measured with a calibrated pH meter (OIV-MA-AS313-15), and density was determined by densimetric analysis (OIV-MA-AS2-01). Free and total sulphur dioxide concentrations (mg L^−1^) were analyzed by iodometric titration (OIV-MA-AS323-04). All analyses of the wines were performed within the same vintage year as the harvest.

### 2.3. Phytochemical Compounds Analysis by LC-MS/MS

The samples’ phytochemical profiles were characterized using four distinct LC-MS/MS analytical methods, previously validated [26,27,28,29,30], performed on an Agilent 1100 HPLC system (Agilent Technologies, Santa Clara, CA, USA). The equipment included a binary pump, an autosampler, a UV detector, and an Agilent Ion Trap 1100 SL mass spectrometer (Agilent Technologies, Santa Clara, CA, USA).

#### 2.3.1. Phenolic Acids and Flavonoids Analysis

The first analytical method was employed for the comprehensive screening of 23 phenolic acids and flavonoids, namely apigenin, caffeic acid, 4-O-caffeoylquinic acid, caftaric acid, chlorogenic acid, p-coumaric acid, ferulic acid, fisetin, gentisic acid, hyperoside, isoquercitrin, kaempferitrin, kaempferol, kaempferol-3-rhamnoside, luteolin, myricetin, patuletin, quercetin, quercitrin, rutoside, sinapic acid, vitexin, and vitexin 2-O-rhamnoside. It was conducted using a binary gradient consisting of methanol and 0.1% aqueous acetic acid (*v*/*v*) at a flow rate of 1 mL/min and an injection volume of 5 μL. Detection was performed via UV at 330 nm (phenolic acids) and 370 nm (flavonoids), with identity confirmation provided by electrospray ionization (ESI) mass spectrometry in negative mode, as previously detailed [26,29,30].

A second analytical method was employed to quantify eight additional phenolics, specifically gallic, protocatechuic, vanillic, and syringic acids, alongside catechin, epicatechin, epigallocatechin and epigallocatechin gallate. This method utilized the same column and mobile phase constituents but applied a modified gradient profile and ESI settings optimized for these specific analytes [26,29,30]. Quantification was performed considering the MS signal following positive identification based on MS spectra against individual analytical standards.

#### 2.3.2. Procyanidin Analysis

Quantification of procyanidins A1, B1, B2, B3, B4, and C1 was performed using a dedicated LC-MS/MS analytical method employing the same instrumentation described for general polyphenolic analysis. Chromatographic separation was achieved at 45 °C using a Zorbax SB-C18 column (100 × times 3.0 µm i.d., 3.5 µm particles). The mobile phase utilized a gradient of 0.1% aqueous acetic acid (A) and methanol (B), at a 1 mL min^−1^ flow rate. The elution profile began at 8% B, increasing linearly to 20% B over 8 min, followed by a 3 min re-equilibration period at the initial conditions. The injection volume for each sample was 5 µL. Detection was carried out in Multiple Reaction Monitoring (MRM) mode using an electrospray ionization (ESI) source in negative polarity. Optimized source parameters included a 3000 V capillary voltage, a 350 °C drying gas temperature with a flow of 12 L min^−1^, and a nebulizer pressure of 60 psi. For quantitative assessment, specific mass transitions were monitored, as previously detailed by Solcan et al. [29], Solcan et al. [31] and Safta et al. [30]. The method demonstrated linearity for all analytes across a concentration range of 0.1–100 µg/mL.

#### 2.3.3. Resveratrol Analysis

For this particular polyphenolic compound belonging to the stilbenoid class, specifically recognized as a type of phytoalexin produced by *Vitis vinifera*, analysis was performed using the same Agilent 1100 HPLC system (Agilent Technologies, Santa Clara, CA, USA) coupled with an Ion Trap SL mass spectrometer via an atmospheric pressure chemical ionization (APCI) source in negative mode. Chromatographic separation was achieved on a Zorbax SB-C18 column (100 × 3.0 × mm i.d., 3.5 μm particles) maintained at 40 °C, using an isocratic mobile phase of 1 mM ammonium acetate and acetonitrile (73:27, *v*/*v*) at a flow rate of 1 mL/min and a 5 μL injection volume. The MS was operated in Multiple Reaction Monitoring (MRM) mode, targeting the transition of *m*/*z* from 227 to 185 with optimized source parameters: 350 °C APCI heater, 60 psi nebulizer pressure, and a 5 L/min dry gas flow at 250 °C. For calibration, *trans*-resveratrol was prepared as a 10 mg mL^−1^ methanolic stock, stored at 4 °C in the dark, and diluted with bidistilled water. Because *cis*-resveratrol is commercially unavailable, it was generated via UV-induced photoisomerization (254 nm for 10 min) with an approximate 90% yield, enabling the construction of linear calibration curves for both *trans* (10.47–837.86 ng mL^−1^, n = 7, R^2^ = 0.993) and *cis* (9.12–730.14 ng mL^−1^, n = 7, R^2^ = 0.995) isomers [27].

#### 2.3.4. Phytochemical Data Interpretation and Analysis

Data were acquired and processed using ChemStation (vB01.03) and DataAnalysis (v5.3) software (Agilent, Santa Clara, CA, USA). For compound authentication, a multi-parameter approach was employed, including the correlation of retention times (R_t_) with analytical standards, UV spectral characteristics, and the verification of diagnostic MS and MS/MS fragmentation patterns, including both precursor and corresponding product ions. For all methods, quantification was performed using external calibration curves [27,30].

### 2.4. Economic Assessment Model

The economic assessment was conducted using a cost modelling approach combining activity-based costing and process-oriented analysis, as commonly applied in food processing and wine production systems [20,32]. This approach allows the decomposition of total cost into material, operational, and capital components.

The total production cost per liter (C_total_) was calculated as:

(1) $$C_{\mathrm{total}}\;=\;C_{\mathrm{wood}}\;+{\;C}_{\mathrm{time}}\;+\;C_{\mathrm{labor}}\;+{\;C}_{\mathrm{energy}}\;+\;C_{\mathrm{equipment}}$$

The wood cost (C_wood_) was estimated based on market prices of oak fragments (3–6 € kg^−1^), resulting in 0.004–0.012 € L^−1^ depending on dosage. The time-dependent cost (C_time_) (0.01–0.03 € L^−1^/day) accounts for storage, capital immobilization, and operational overhead, which are recognized as major cost drivers in wine production systems [33,34]. Labor cost (C_labor_) was estimated at 0.01–0.02 € L^−1^, while energy cost (Cenergy) for ultrasound-assisted treatments ranged from 0.02 to 0.05 € L^−1^. Equipment cost (Cequipment) includes amortization of technological investments, following standard cost allocation practices.

For comparison, the cost of traditional barrel ageing (6–12 months) was estimated using a simplified techno-economic approach based on literature data. The total cost includes barrel depreciation, storage (time-related costs), and labor.

Barrel depreciation is the main contributor, as oak barrels (≈500–1000 €) are used for a limited number of cycles, resulting in ~0.8–1.5 € L^−1^. Storage and capital immobilization during ageing contribute ~0.8–2.5 € L^−1^, while labor and maintenance operations add ~0.5–2.0 € L^−1^. Overall, the total cost of barrel ageing is estimated at 2.4–6.1 € L^−1^, depending on ageing duration and winery practices, consistent with reported wine production cost structures [34,35,36].

- Cost–Efficiency Evaluation Based on Phenolic Extraction

The cost-efficiency indicator (E) was calculated for each experimental variant according to the following equation:

(2) $$E=C_{\mathrm{total}}/\mathrm{Gallic}\;\mathrm{acid}$$

where E (€ mg^−1^) represents the cost per unit of gallic acid, C_total_ is the production cost (€ L^−1^), and gallic acid is expressed in mg L^−1^.

This indicator expresses the cost required to obtain one unit (mg) of extracted phenolic compound, enabling direct comparison between treatments with different extraction yields and processing costs. It is based on cost-to-performance metrics commonly applied in techno-economic assessments, where process efficiency is normalized to a target compound or functional output. Similar normalization approaches are commonly employed in process intensification and extraction studies, where performance is expressed as the ratio between operational cost and target compound yield, facilitating objective evaluation and optimization of process configurations [11,37]. Gallic acid was selected as a representative marker of oak-derived phenolics because it originates primarily from wood tannin hydrolysis and showed the most pronounced variation across treatments.

### 2.5. Statistical Analysis

Statistical analyses were performed using XLSTAT–Basic (Lumivero, Denver, CO, USA). Prior to inferential testing, exploratory data analysis was conducted to assess data distribution and identify potential outliers. Normality was assessed using the Shapiro–Wilk test. Differences among experimental variants were evaluated using one-way ANOVA. Homogeneity of variances was tested using the Brown–Forsythe test. When this assumption was violated, Welch’s ANOVA was applied as a robust alternative. Effect sizes were calculated using partial eta-squared (ηp^2^). When significant effects were identified, Tukey’s HSD test was used for pairwise comparisons.

Principal Component Analysis (PCA) was applied to standardized variables using the correlation matrix to explore multivariate structure and reduce dimensionality. Component retention was based on the Kaiser criterion (eigenvalues > 1), scree plot inspection, and cumulative explained variance.

Statistical significance was set at *p* < 0.05.

## 3. Results

### 3.1. Physicochemical Parameters

The results of the physicochemical analyses of the experimental variants are presented in Table 2. All measured parameters fell within the limits established by the International Organisation of Vine and Wine (OIV), confirming that none of the applied treatments adversely affected the fundamental quality or regulatory compliance of the wines [25]. As all variants originated from the same base wine, the observed differences can be attributed primarily to the applied maturation strategies, namely the addition of oak wood fragments (granules or chips), variations in wood dosage and contact time, and the application of ultrasound treatment, which may have accelerated mass transfer and ageing-related processes. Most physicochemical parameters were not significantly affected by the different oak fragments.

The variations in total acidity and pH across the oak-treated wine samples and the control are attributable to the extraction of organic acids and hydrolysable tannins from oak wood [31]. The pH values remained within a narrow range (3.15–3.37) and showed no statistically significant differences (*p* > 0.05) among the maturation treatments (Table 2). This stability suggests that neither the addition of oak wood fragments nor the interaction between wood contact and ultrasound treatment substantially altered the acid–base equilibrium of the matrix.

In contrast, total acidity exhibited significant variability across the experimental variants (*p* < 0.05), with values ranging from 7.03 ± 0.08 g L^−1^ (V12) to 7.95 ± 0.20 g L^−1^ (V31). The statistical analysis revealed a large effect size (ŋp^2^ = 0.603) associated with treatment effects, indicating that the observed variability is strongly influenced by the applied maturation conditions. Wines matured with oak chips generally displayed lower total acidity (e.g., V12 at 7.03 g L^−1^), whereas those treated with oak granules, such as V31, showed significantly higher levels (7.95 g L^−1^).

These differences are likely related to the extraction kinetics and surface area-to-volume ratios of the wood fragments. Oak granules, characterized by smaller particle sizes, facilitate a more rapid and extensive release of organic acids and hydrolyzable tannins [38,39]. Conversely, the larger dimensions of oak chips result in a more gradual extraction, limiting the total acidic contribution. The application of ultrasound further enhanced mass transfer processes, as indicated by a significant treatment effect (*p* < 0.05, ŋp^2^ = 0.251), enhancing compound extraction kinetics and contributing to the observed variability in total acidity without compromising the high buffering capacity of the wine matrix [40].

Volatile acidity (VA) is a pivotal quality parameter in white wines, with even slight increments capable of altering sensory perception and perceived wine stability. The relatively limited variation in VA among experimental variants underscores the modulatory effects of oak dosage and oxygen availability during post-fermentative maturation. Wines subjected to higher oak wood dosages, particularly variants V13–V18 and V26–V31, manifested marginally elevated VA values (0.38–0.39 g L^−1^ acetic acid), relative to the control.

These observations indicate that an increased wood contact area, combined with enhanced oxygen transfer, may promote oxidative reactions at the wine-wood interface, thereby facilitating acetic acid formation via ethanol oxidation under micro-oxygenation conditions. Controlled oxygen exposure is well established as a key factor in shaping volatile profiles during oak ageing, as oxygen transfer influences both oxidation kinetics and the evolution of volatile compounds within wine matrices [41,42]. Although statistically significant differences were observed (*p* < 0.05), the magnitude of variation remained relatively small, suggesting limited practical impact on wine quality.

Oxidative mechanisms are closely linked to acetic acid formation and SO_2_ consumption, making SO_2_ dynamics a key consideration alongside volatile acidity. Free and total SO_2_ are critical indicators of oxidative stability and antimicrobial protection in white wines [16]. In this study, ultrasound-treated wines (V36—40 mg L^−1^) exhibited lower free and total SO_2_ compared to the control (V0) and wines matured with oak fragments alone.

Although these differences were statistically significant (*p* < 0.05), their absolute magnitude remained moderate. The observed decrease can be attributed to accelerated redox reactions induced by ultrasonic cavitation. Ultrasound generates localized high-energy conditions that enhance mass transfer and promote the formation of reactive species, promoting phenolic oxidation and carbonyl formation (e.g., acetaldehyde), which reversibly bind SO_2_. In addition, ultrasound may increase the extraction of wood-derived phenolics, thereby increasing the pool of redox-active compounds and further contributing to SO_2_ consumption [43]. These results indicate that oak characteristics and ultrasound jointly influence oxidative dynamics, with observed changes in total and volatile acidity, pH, and SO_2_ reflecting interconnected redox processes within the wine matrix. This interdependence between physico-chemical parameters and sulfur dioxide dynamics underscores the need for a thorough exploration of the phenolic profile, as the compounds extracted from the wood not only define the wine’s sensory footprint but also play a critical role in regulating its oxidative stability.

### 3.2. Phenolic Compounds

Oak-derived phenolics (ellagitannins, phenolic acids, and lignin-derived phenols) are extracted during maturation and influence astringency, color stability, and oxidative reactions via redox processes and acetaldehyde-mediated condensation [16,44]. Toasting alters this composition by degrading lignin and hemicellulose, increasing volatile compounds such as vanillin, furfural, and oak lactones while reducing ellagitannins, thereby shifting the balance toward greater aromatic complexity [45].

#### 3.2.1. Flavan-3-Ols

The extraction of oak-derived constituents resulted in higher phenolic concentrations in wood-treated samples compared to controls, aligned with established maturation trends. However, the technological relevance of these increases, particularly in white wine matrices, requires a nuanced evaluation. Ultrasound treatment demonstrated compositional profiles comparable to conventional maturation, proving to be a time-efficient alternative, as previously observed in red wines [46,47]. Catechin and epicatechin concentrations exhibited a progressive decline (up to 34%), typical of the oxidative polymerization reactions inherent to maturation. Notably, the combination of oak and ultrasound appeared to moderate this loss, with reductions limited to 28% [48]. This may indicate that ultrasound accelerates the initial extraction phase, leading to faster saturation of the matrix with extracted phenolics and partially limiting subsequent oxidative losses [49]. While statistically significant (*p* < 0.05), the practical relevance of this 6% difference is more evident in process efficiency (achieving stability in 10–20 days) rather than in major compositional differences [24].

The detection of procyanidins B1–B4 (0.02–0.32 mg L^−1^) highlights the limited sensory relevance of statistically significant differences. While variants V5, V8 and V20 showed intensified extraction due to ultrasound-enhanced mass transfer, these concentrations remain significantly lower than those found in red wines, where ultrasound is used to disrupt grape skin cell walls [6]. In our white wine matrix, the lack of solid grape material limits the ultrasound’s impact on the wood–wine interface. Therefore, the magnitude of the treatment effect, reflected in large effect sizes for the extraction kinetics of procyanidins B1–B4 (ŋp^2^ = 0.210–0.362), supports the use of ultrasound as an effective tool for process optimization, without fundamentally altering the characteristic phenolic profile of white wines.

Procyanidins are condensed tannins composed of catechin and epicatechin units and are more characteristic of red wines, where long skin maceration facilitates their extraction from grape skins and seeds [50]. Notably, certain treatments, especially in variants V5, V8, and V20, exhibited slight elevations in procyanidin B1–B4 concentrations compared with the control (V0). This suggests that intensified extraction conditions, such as increased oak granule surface area, higher doses of wood, or enhanced mass transfer mechanisms (e.g., ultrasound), can enhance the release of oligomeric flavan-3-ols even in white wine matrices [10].

#### 3.2.2. Phenolic Acids

The concentration of syringic acid, a classic marker of lignin degradation, increased significantly across all oak treatments, consistent with enhanced extraction from oak material. Gallic acid exhibited a strong response to treatment conditions, reflecting both the extraction processes and hydrolysis of gallotannins (Table 3). This trend mirrors the behaviour observed for catechin and epicatechin.

Gallic acid levels rose from 1.54 mg L^−1^ (V0) to a peak of 4.41 mgL^−1^ in V20 (2 mg L^−1^ medium-toasted granules, 20 days). While this three-fold increase is statistically significant (*p* < 0.05), the strength of the finding is underscored by a large effect size (ŋp^2^ = 0.958) for the physical form of the oak. This suggests that the transition from chips to granules is not merely a minor change but a primary driver of phenolic release, facilitating faster diffusion through an expanded solid–liquid interface [51]. This is consistent with previously reported surface area effects by Sánchez-Gómez et al. [45], who demonstrated that the smaller size of oak fragments (chips vs. larger fragments such as staves) significantly influences the extraction rate of low-molecular-weight phenolic acids [52]. The elevation in gallic acid is traditionally attributed to the hydrolysis of gallotannins; however, our data suggests that ultrasound-assisted extraction (UAE) further accelerates the hydrolysis-extraction equilibrium. While studies on white wines matured with oak fragments [10] show similar qualitative increases, the technological relevance of our findings lies in the time-compression achieved.

This pronounced increase can be attributed to enhanced extraction of wood-derived phenolic compounds facilitated by prolonged contact with toasted oak material, as well as to the partial hydrolysis of gallotannins, leading to the release of free gallic acid into the wine matrix [53]. Similar findings have been reported in red wines, where significant increases in gallic acid and other low-molecular-weight phenolic acids were observed following maturation with oak chips, oak staves or barrels, compared with control wines [45,53,54]. This trend is also evident in white wines subjected to oak treatments. For instance, a study conducted in China on Longyan grapes evaluated wines aged with oak from three different regions (Yanshan, American and French) at concentrations of 2, 4 and 6 g L^−1^. The results demonstrated a clear increase in gallic acid across all oak-treated wines, with the magnitude of the increase being largely consistent among the different oak origins, indicating that oak contact enhances phenolic extraction irrespective of wood provenance [55].

#### 3.2.3. Hydroxycinnamic Acids

White wines contain hydroxycinnamic acids, notably caftaric, caffeic and *p*-coumaric acids, which represent the principal non-flavonoid phenolics originating from grape tissues. These compounds are found either in their free form or esterified with tartaric acid, and they play a significant role in oxidation processes and the evolution of color during wine maturation [56,57]. Hydroxycinnamic acids generally exhibited limited variability in response to treatment intensity, although statistically significant differences were observed for certain compounds.

Caftaric acid is the major grape-derived hydroxycinnamic acid in white wines, formed as a tartaric ester of caffeic acid during early berry processing, particularly after crushing, where enzymatic oxidation via polyphenol oxidase contributes to its formation. In the present study, its concentration remained high and relatively stable across all treatments (36.3–38.9 mg L^−1^), with only minor but statistically significant variation (ηp^2^ = 0.083), indicating limited technological relevance. Neither ultrasound treatment nor oak contact induced substantial changes in its levels, suggesting a low sensitivity to the applied post-fermentative interventions under the studied conditions. Caftaric acid is important as the primary substrate for oxidative browning in white wines, being converted to caftaric quinones during enzymatic oxidation, which drives color evolution [56,57].

Caffeic acid concentrations ranged narrowly between 1.11 mg L^−1^ (V2) and 1.37 mg L^−1^ (V14), with V0 and V00 showing comparable levels (1.13 mg L^−1^ and 1.20 mg L^−1^, respectively), confirming that neither oak fragment addition nor ultrasound application substantially altered its concentration. Even in samples showing intensified extraction of oak-derived compounds (V20 or V23), caffeic acid remained within a similar range, 1.18–1.24 mg L^−1^, confirming its overall stability across treatments.

A similar pattern was observed for *p*-coumaric acid, which ranged between 0.10 mg L^−1^ (V12) and 0.24 mg L^−1^ (V5, V16), while V0 and V00 recorded 0.22 mg L^−1^ and 0.18 mg L^−1^, respectively. The relatively low dispersion of values (coefficient of variation <10–15% across most treatments) suggests that these compounds, mainly originating from grape hydroxycinnamoyl tartaric esters, had already reached extraction equilibrium during fermentation and were not substantially influenced by post-fermentative technological interventions. These observations are consistent with earlier reports indicating that hydroxycinnamic acids, including caffeic and *p*-coumaric acids, undergo only limited modifications during oak maturation. Although trace amounts may be contributed by the wood, oak is not a major source of these compounds and their concentrations tend to remain relatively constant during maturation, irrespective of whether barrels, chips or staves are used. Published data generally show that total hydroxycinnamic acid derivatives in red wines remain within similar ranges throughout maturation, typically around 60–130 mg L^−1^, supporting the view that these phenolics are only marginally influenced by wood contact compared to more extraction-sensitive compounds [58].

#### 3.2.4. Stilbenes

Stilbenes exhibited moderate but noteworthy variation across the different maturation treatments. *Trans*-resveratrol concentrations ranged from 0.08 mg L^−1^ in samples such as V17 and V33 to 0.19 mg L^−1^ in V16, while *cis*-resveratrol showed a broader distribution, reaching a maximum of 0.34 ± 0.01 mgL^−1^ in V20. These values are consistent with those typically reported for white wines, where resveratrol concentrations are generally low due to limited skin contact during vinification. For example, average *trans-* and *cis*-resveratrol concentrations of approximately 0.12 mg L^−1^ have been reported in white wines from Catalonia [59], while broader surveys indicate mean values around 0.13 mg L^−1^ and maxima up to ~0.82 mg L^−1^ depending on grape variety and processing conditions [60]. The concentrations obtained in the present study, therefore, fall within the expected range for white wines. Slight increases in *cis*-resveratrol in some ultrasound-treated samples may be associated with *trans-cis* isomerization induced by acoustic cavitation, which generates localized temperature and pressure gradients, promoting structural conversion without significantly altering the total stilbene content.

#### 3.2.5. Lignin-Derived Phenolics/Coumarins

Lignin-derived phenolics, especially esculetin and syringaldehyde, showed significant increases in response to alternative maturation. In the control samples, esculetin levels were very low or below the detection limit, whereas treatments such as V3 (0.84 mg L^−1^) and V15 (0.72 mg L^−1^) showed clear increases, reflecting the release of coumarin compounds from oak. Syringaldehyde concentrations were consistently higher across treatments, reaching 1.44 ± 0.01 mg L^−1^ in V9 and 1.33 mg L^−1^ in V21, compared with 1.13 mg L^−1^ (V0) and 1.20 mg L^−1^ (V00). These results are consistent with previous studies on oak maturation, which reported that syringaldehyde and other lignin-derived aldehydes increase significantly in wines or vinegars aged in oak compared with controls without wood contact, confirming that oak is a key source of these phenolics [61].

**Figure 2:**
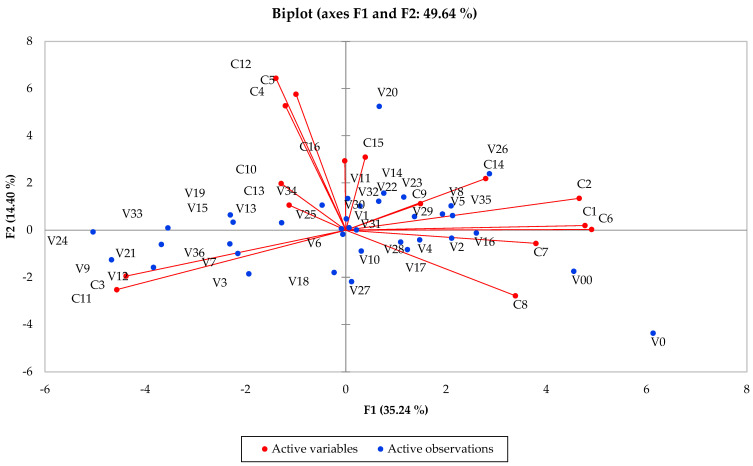
Principal component analysis (PCA) of phenolic compounds identified in wine samples, showing the distribution of variables along the first two principal components (F1 and F2), which explain 49.64% of the total variance. The variables are coded as follows: C1—epicatechin; C2—catechin; C3—syringic acid; C4—gallic acid; C5—protocatechuic acid; C6—procyanidin B1; C7—procyanidin B2; C8—procyanidin B3; C9 -procyanidin B4; C10—esculetin; C11—syringaldehyde; C12—caftaric acid; C13—caffeic acid; C14—*p*-coumaric acid; C15—*trans*-resveratrol; and C16—*cis*-resveratrol. The plot illustrates the distribution pattern of the studied phenolic compounds based on their loadings on the principal components.

The principal component analysis (PCA) (Figure 2) explained 49.64% of the total variance, indicating that approximately half of the multivariate phenolic variability is captured by the first two principal components. This reflects a moderately complex dataset structure, with a substantial proportion of variability distributed across higher-order dimensions not represented in the two-dimensional projection. The distribution of samples along F1 reflects differences primarily associated with phenolic compounds that were also identified as significant in the univariate analysis (Table 3), including catechin, epicatechin and procyanidin B1, which showed higher loadings on the positive side of the axis. In contrast, syringic acid and other phenolic acids were positioned on the negative side of F1. This separation reflects compositional differences among samples associated with treatment conditions. Samples located on the positive side of F1 (e.g., V0 and V16) are associated with a higher relative contribution of flavan-3-ols and procyanidins, whereas samples positioned on the negative side (e.g., V9, V12 and V24) are more closely related to phenolic acid profiles.

The second principal component (F2) captured additional variability associated with gallic acid, protocatechuic acid and resveratrol isomers. However, in agreement with the ANOVA results, which indicated lower effect sizes for stilbenes compared to flavan-3-ols and phenolic acids, separation along this axis was less pronounced. The loading structure revealed coherent grouping patterns, with procyanidins (C6–C9) clustering together and phenolic acids (C3–C5) forming a distinct group, reflecting their correlated variation across samples.

Regarding sample distribution, most variants were located near the center of the PCA plot, in agreement with the moderate effect sizes observed in ANOVA for several parameters, indicating broadly similar phenolic profiles across treatments. A limited number of samples (e.g., V20 and V26) were positioned further from the origin, reflecting more distinct phenolic signatures in relation to the measured variables. Overall, PCA highlights that sample differentiation is primarily driven by variations in flavan-3-ols and phenolic acids, consistent with the multivariate structure observed in the dataset.

### 3.3. Economic Sustainability Assessment

#### 3.3.1. Effect of Contact Time

In our study, the duration of oak fragment contact had a more substantial influence on phenolic accumulation than the amount of wood added. Across treatments, wines aged with oak fragments for 20 days showed consistently higher levels of gallic acid, syringaldehyde, and other lignin-derived phenolics than those aged for only 10 days, indicating that extended contact time enhances extraction efficiency. This observation aligns with earlier research showing that wood-derived phenolics accumulate progressively over time during contact with oak chips, reflecting diffusion-limited mass transfer rather than instantaneous release of all extractable compounds [10].

Comparisons between the control samples further clarify these mechanisms. The untreated control (V0) maintained baseline phenolic concentrations characteristic of the wine matrix, whereas V00 (wine exposed to ultrasound alone) exhibited only minor increases in gallic acid and procyanidins. These modest changes suggest that ultrasound can slightly enhance the release of endogenous wine phenolics, possibly by facilitating breakdown of weak interactions or accelerating desorption from suspended solids, but it does not introduce oak-specific compounds or match the impact of direct oak contact. This is consistent with literature reporting that ultrasound, while capable of accelerating early extraction steps through cavitation and micro-mixing, generally does not increase final phenolic equilibria in wine systems in the absence of a substantive extraction source such as wood chips or staves [10,46].

The results also imply that equilibrium between wine and wood-derived phenolics was not reached within 10 days, particularly for larger molecules like procyanidins. Similar biphasic extraction behaviour has been documented in oak contact studies, where low-molecular-weight phenolic compounds are released rapidly at early stages, followed by a slower phase of accumulation for higher-molecular-weight constituents as diffusion through the wood matrix becomes rate-limiting [45]. The accelerated extraction observed in our 5 L fragment system likely reflects the greater surface area and shorter diffusion paths provided by small wood fragments compared with traditional staves, enabling a more rapid approach toward equilibrium without requiring prolonged ageing.

#### 3.3.2. Economic Sustainability

The economic sustainability of the tested maturation strategies was evaluated using a comparative cost analysis combining experimental results with literature data (Table 4). Processing time clearly emerged as the main cost driver, while raw material costs were relatively low.

Barrel ageing showed the highest costs (2.4–6.1 € L^−1^), consistent with literature values, where barrel depreciation alone is around 0.5–0.6 € L^−1^, excluding storage and labor [19,20]. The prolonged maturation period (typically 6–12 months) generates substantial opportunity costs due to limited tank turnover and delayed commercialization [62,63]. In contrast, oak fragment technologies (chips and granules) significantly reduced production costs to 0.11–0.32 € L^−1^ (10 days) and 0.23–0.63 € L^−1^ (20 days). Literature results are consistent with previous studies showing that oak alternatives are substantially less expensive than traditional barrels and can significantly reduce production costs while maintaining similar compositional characteristics [64,65]. The reduction in production costs is mainly driven by the significant decrease in maturation time, from several months in barrel ageing to only days when using oak fragments. This shortening of the process reduces storage requirements, limits capital immobilization, and improves production turnover. Previous studies have shown that oak alternatives enable faster extraction of phenolic and aromatic compounds, primarily due to their higher surface area-to-volume ratio, which enhances mass transfer between wood and wine [66]. Short-duration treatments (10 days) were more cost-efficient than 20-day treatments, confirming that time-dependent costs increase disproportionately with process duration. This is consistent with a study made in Spain on Monastrell grapes, which showed that most oak-derived compounds are extracted in the early stages, with diminishing returns over time. As a result, prolonged maturation reduces economic efficiency without clear quality benefits [67].

#### 3.3.3. Cost–Efficiency Analysis of Phenolic Extraction

The integration of economic and compositional data enabled evaluation of maturation strategies based on cost efficiency (€ mg^−1^ gallic acid), providing a more relevant indicator than cost or extraction alone (Table 5). This approach reflects current trends in techno-economic analysis, where performance is related to functional outputs [11]. The results show that economic sustainability depends primarily on extraction efficiency rather than absolute phenolic content.

Ultrasound-assisted treatments (V25–V36) showed the highest efficiency (<0.03 € mg^−1^), combining moderate–high gallic acid levels (2.05–3.28 mg L^−1^) with very low costs (0.05–0.07 € L^−1^). This confirms that ultrasound enhances mass transfer and accelerates extraction while reducing processing time [11,41].

Short-duration treatments (10 days) formed the second efficiency group (0.05–0.07 € mg^−1^), supporting previous findings that phenolic extraction increases rapidly during early contact with oak and evolves with time [68]. In contrast, 20-day treatments were less efficient (>0.10 € mg^−1^), indicating diminishing returns despite higher phenolic concentrations. Compared to these approaches, barrel ageing yields similar phenolic levels but at much higher cost (2.4–6.1 € L^−1^), mainly due to long storage and labour requirements [19]. Consequently, its cost-efficiency (~1.0 € mg^−1^) is markedly lower than all experimental variants.

The results demonstrate that optimal performance is achieved by maximizing extraction per unit cost rather than absolute extraction. Ultrasound-assisted treatments are the most economically sustainable option, followed by short-term treatments, while long-term maturation and barrel ageing remain economically inefficient.

### 3.4. Study Limitations

The analytical method employed in this study was previously validated on vegetal matrices rather than specific wine matrices. Consequently, the referenced LOD/LOQ values were established for the former. We acknowledge that the complex wine matrix—specifically its ethanol content, organic acids, and polysaccharides—may influence liquid chromatography retention and electrospray ionization efficiency, and the lack of matrix-specific validation constitutes a methodological limitation of the current analytical workflow.

## 4. Conclusions

This study indicates that the combination of oak wood fragments and ultrasound-assisted processing offers a highly effective alternative for accelerating the extraction of phenolic compounds in white winemaking. The results demonstrate that the physical form of the wood is a primary driver of extraction efficiency; specifically, oak granules consistently outperformed other formats, likely due to the increased surface-to-volume ratio and enhanced solid–liquid contact. While contact time emerged as a critical parameter for compound accumulation in conventional alternative maturation, the application of ultrasound (15 min at 35 kHz) served as a powerful process intensification tool. It significantly accelerated mass transfer and the release of oak-derived phenolics, drastically compressing the extraction timeframe compared to conventional approaches. Crucially, as evidenced by multivariate analysis, these enhanced extraction yields were achieved without compromising the fundamental identity and typicity of the base wine. From a preliminary economic and environmental perspective, the results suggest that replacing traditional barrel maturation with oak fragment-based treatments could lead to substantial cost reductions by minimizing processing time and improving production throughput. The cost-efficiency analysis indicates that ultrasound-treated variants provided an optimal balance between phenolic yield and economic viability, achieving highly competitive extraction costs (<0.03 € mg^−1^ gallic acid). Furthermore, this approach aligns directly with resource efficiency goals by minimizing wood consumption, reducing processing time, and lowering storage-related capital immobilization. Overall, these findings provide a kinetic basis for integrating oak fragments and ultrasound technology into white wine production to improve process flexibility. However, while this strategy strongly supports current trends in oenological innovation, its broader implications for industrial sustainability and circular economy principles warrant further investigation through pilot-scale validation and comprehensive life-cycle assessments.

## Figures and Tables

**Table 1:** Experimental variants.

Sample Code	Commercial Designation	Toasting Level	Dose g L^−1^	Time	Treatments
V0	-	-	-	-	Alternative maturation using oak wood fragments
V1	Granules	fresh	1	10 days	
V2	Granules	light			
V3	Granules	medium			
V4	Chips	fresh			
V5	Chips	light			
V6	Chips	medium			
V7	Granules	fresh		20 days	
V8	Granules	light			
V9	Granules	medium			
V10	Chips	fresh			
V11	Chips	light			
V12	Chips	medium			
V13	Granules	fresh	2	10 days	
V14	Granules	light			
V15	Granules	medium			
V16	Chips	fresh			
V17	Chips	light			
V18	Chips	medium			
V19	Granules	fresh		20 days	
V20	Granules	light			
V21	Granules	medium			
V22	Chips	fresh			
V23	Chips	light			
V24	Chips	medium			
V00	-	-	-	15 min	Oak wood fragments + ultrasounds
V25	Granules	fresh	1		
V26	Granules	light			
V27	Granules	medium			
V28	Chips	fresh			
V29	Chips	light			
V30	Chips	medium			
V31	Granules	fresh	2		
V32	Granules	light			
V33	Granules	medium			
V34	Chips	fresh			
V35	Chips	light			
V36	Chips	medium			

**Table 2:** Physicochemical Characterization of Wines Subjected to Alternative Maturation Treatments.

Sample Code	TA (g L^−1^)	VA (g L^−1^)	pH	AS (% Vol. Alc.)	D	RS (g L^−1^)	Total SO_2_ (mg L^−1^)	Free SO_2_ (mg L^−1^)
V0	7.49 ± 0.02 ^c^	0.36 ± 0.05	3.21 ± 0.00	14.4 ± 0.02 ^bc^	0.9916 ± 0.02	0.1 ± 0.02 ^i^	134 ± 0.15 ^a^	48 ± 0.08 ^a^
V1	7.49 ± 0.10 ^bc^	0.36 ± 0.00	3.21 ± 0.24	14.4 ± 0.00 ^abc^	0.9915 ± 0.01	0.1 ± 0.00 ^i^	127 ± 0.01 ^d^	46 ± 0.00 ^cd^
V2	7.49 ± 0.20 ^bc^	0.37 ± 0.01	3.21 ± 0.00	14.5 ± 0.06 ^abc^	0.9915 ± 0.00	0.1 ± 0.00 ^i^	128 ± 0.15 ^f^	45 ± 0.16 ^de^
V3	7.49 ± 0.09 ^bc^	0.39 ± 0.00	3.17 ± 0.03	14.5 ± 0.05 ^abc^	0.9917 ± 0.01	0.1 ± 0.00 ^j^	129 ± 0.02 ^b^	48 ± 0.11 ^a^
V4	7.49 ± 0.16 ^bc^	0.37 ± 0.01	3.21 ± 0.05	13.9 ± 0.20 ^abc^	0.9915 ± 0.17	0.4 ± 0.20 ^j^	127 ± 0.07 ^b^	46 ± 0.05 ^a^
V5	7.34 ± 0.14 ^abc^	0.35 ± 0.18	3.20 ± 0.06	14.5 ± 0.11 ^bcd^	0.9918 ± 0.10	0.0 ± 0.08 ^fg^	125 ± 0.00 ^c^	49 ± 0.04 ^bc^
V6	7.49 ± 0.20 ^bc^	0.32 ± 0.10	3.20 ± 0.07	14.4 ± 0.07 ^abc^	0.9915 ± 0.05	0.3 ± 0.05 ^hi^	129 ± 0.09 ^c^	47 ± 0.08 ^cd^
V7	7.49 ± 0.17 ^bc^	0.32 ± 0.05	3.21 ± 0.06	14.3 ± 0.10 ^abc^	0.9916 ± 0.01	0.4 ± 0.21 ^cde^	125 ± 0.02 ^c^	45 ± 0.06 ^de^
V8	7.49 ± 0.10 ^abc^	0.31 ± 0.01	3.16 ± 0.01	14.3 ± 0.05 ^bcd^	0.9918 ± 0.16	0.7 ± 0.03 ^ab^	129 ± 0.08 ^c^	46 ± 0.10 ^ab^
V9	7.80 ± 0.16 ^ab^	0.30 ± 0.04	3.25 ± 0.04	14.6 ± 0.01 ^ab^	0.9913 ± 0.02	0.0 ± 0.15 ^cde^	127 ± 0.00 ^f^	45 ± 0.11 ^e^
V10	7.49 ± 0.06 ^bc^	0.33 ± 0.02	3.18 ± 0.05	14.5 ± 0.20 ^abc^	0.9918 ± 0.14	0.7 ± 0.04 ^cde^	126 ± 0.13 ^f^	46 ± 0.02 ^e^
V11	7.19 ± 0.00 ^a^	0.33 ± 0.17	3.18 ± 0.20	13.8 ± 0.00 ^cd^	0.9919 ± 0.06	0.4 ± 0.05 ^bcd^	126 ± 0.02 ^e^	45 ± 0.00 ^e^
V12	7.03 ± 0.08 ^a^	0.33 ± 0.01	3.2 ± 0.04	13.5 ± 0.07 ^cd^	0.9915 ± 0.07	0.5 ± 0.11 ^abc^	129 ± 0.00 ^e^	44 ± 0.18 ^de^
V13	7.49 ± 0.21 ^abc^	0.38 ± 0.04	3.24 ± 0.00	14.4 ± 0.03 ^ab^	0.9915 ± 0.00	0.0 ± 0.02 ^k^	128 ± 0.10 ^e^	43 ± 0.10 ^e^
V14	7.49 ± 0.20 ^bc^	0.35 ± 0.18	3.24 ± 0.10	14.6 ± 0.10 ^bcd^	0.9916 ± 0.03	0.1 ± 0.00 ^i^	129 ± 0.09 ^d^	45 ± 0.05 ^de^
V15	7.49 ± 0.12 ^bc^	0.38 ± 0.20	3.19 ± 0.11	14.5 ± 0.08 ^abc^	0.9916 ± 0.03	0.1 ± 0.04 ^ij^	130 ± 0.10 ^d^	49 ± 0.20 ^cde^
V16	7.65 ± 0.04 ^bc^	0.38 ± 0.04	3.35 ± 0.05	14.4 ± 0.05 ^bcd^	0.9917 ± 0.02	0.1 ± 0.00 ^hi^	129 ± 0.04 ^d^	42 ± 0.20 ^f^
V17	7.34 ± 0.10 ^bc^	0.34 ± 0.00	3.20 ± 0.01	14.4 ± 0.10 ^bcd^	0.9916 ± 0.06	0.1 ± 0.17 ^gh^	125 ± 0.25 ^hi^	41 ± 0.09 ^f^
V18	7.49 ± 0.05 ^bc^	0.39 ± 0.00	3.20 ± 0.02	14.6 ± 0.19 ^bcd^	0.9915 ± 0.02	0.1 ± 0.02 ^i^	129 ± 0.04 ^j^	46 ± 0.11 ^fg^
V19	7.65 ± 0.20 ^abc^	0.28 ± 0.11	3.22 ± 0.04	14.5 ± 0.06 ^ab^	0.9918 ± 0.05	0.1 ± 0.00 ^bcd^	125 ± 0.00 ^k^	42 ± 0.20 ^h^
V20	7.65 ± 0.20 ^bc^	0.29 ± 0.05	3.24 ± 0.06	14.4 ± 0.08 ^abc^	0.9915 ± 0.03	0 ± 0.01 ^ef^	126 ± 0.10 ^i^	42 ± 0.09 ^de^
V21	7.65 ± 0.03 ^abc^	0.34 ± 0.01	3.17 ± 0.07	14.6 ± 0.10 ^bcd^	0.9915 ± 0.17	0.2 ± 0.00 ^cde^	123 ± 0.13 ^g^	42 ± 0.00 ^f^
V22	7.49 ± 0.11 ^abc^	0.32 ± 0.05	3.18 ± 0.02	14.3 ± 0.04 ^bcd^	0.9918 ± 0.04	0.6 ± 0.10 ^ef^	122 ± 0.05 ^g^	43 ± 0.07 ^f^
V23	7.80 ± 0.02 ^abc^	0.19 ± 0.01	3.37 ± 0.20	14.5 ± 0.08 ^cd^	0.9921 ± 0.00	0.1 ± 0.00 ^a^	125 ± 0.10 ^f^	44 ± 0.06 ^fg^
V24	7.65 ± 0.09 ^abc^	0.30 ± 0.09	3.23 ± 0.00	14.4 ± 0.02 ^abc^	0.9917 ± 0.02	0.1 ± 0.00 ^fg^	128 ± 0.02 ^g^	43 ± 0.08 ^fg^
V00	7.49 ± 0.00 ^bc^	0.30 ± 0.05	3.22 ± 0.10	14.4 ± 0.20 ^abc^	0.9917 ± 0.16	0.1 ± 0.01 ^hi^	127 ± 0.04 ^a^	41 ± 0.02 ^a^
V25	7.65 ± 0.07 ^bc^	0.30 ± 0.20	3.25 ± 0.16	14.4 ± 0.11 ^ab^	0.9915 ± 0.02	0.2 ± 0.01 ^cde^	124 ± 0.06 ^a^	42 ± 0.10 ^i^
V26	7.49 ± 0.06 ^bc^	0.35 ± 0.02	3.20 ± 0.12	14.6 ± 0.02 ^ab^	0.9919 ± 0.07	0.1 ± 0.02 ^k^	125 ± 0.16 ^b^	45 ± 0.03 ^j^
V27	7.65 ± 0.08 ^abc^	0.37 ± 0.00	3.23 ± 0.08	14.6 ± 0.00 ^ab^	0.9914 ± 0.10	0.1 ± 0.00 ^bcd^	129 ± 0.11 ^b^	46 ± 0.20 ^j^
V28	7.49 ± 0.00 ^bc^	0.34 ± 0.10	3.17 ± 0.05	14.4 ± 0.00 ^e^	0.9922 ± 0.15	0.01 ± 0.01 ^cde^	127 ± 0.19 ^hi^	44 ± 0.17 ^k^
V29	7.80 ± 0.10 ^a^	0.34 ± 0.14	3.21 ± 0.04	14.6 ± 0.04 ^de^	0.9911 ± 0.02	0.1 ± 0.01 ^ij^	126 ± 0.06 ^c^	45 ± 0.20 ^gh^
V30	7.80 ± 0.03 ^bc^	0.34 ± 0.05	3.18 ± 0.00	14.4 ± 0.16 ^cd^	0.9913 ± 0.00	0.4 ± 0.02 ^gh^	126 ± 0.18 ^d^	44 ± 0.19 ^k^
V31	7.95 ± 0.20 ^abc^	0.33 ± 0.11	3.32 ± 0.10	14.6 ± 0.02 ^ab^	0.9916 ± 0.04	0.1 ± 0.00 ^bcd^	123 ± 0.11 ^h^	42 ± 0.00 ^i^
V32	7.49 ± 0.07 ^abc^	0.28 ± 0.23	3.21 ± 0.16	14.2 ± 0.03 ^ab^	0.9915 ± 0.10	0.4 ± 0.03 ^k^	124 ± 0.04 ^d^	43 ± 0.04 ^i^
V33	7.65 ± 0.20 ^abc^	0.33 ± 0.00	3.23 ± 0.07	14.6 ± 0.00 ^ab^	0.9916 ± 0.04	0.1 ± 0.00 ^de^	121 ± 0.08 ^f^	42 ± 0.06 ^i^
V34	7.34 ± 0.05 ^abc^	0.34 ± 0.03	3.17 ± 0.05	14.1 ± 0.10 ^a^	0.9916 ± 0.20	0.7 ± 0.01 ^fg^	120 ± 0.20 ^g^	42 ± 0.08 ^i^
V35	7.34 ± 0.10 ^abc^	0.35 ± 0.13	3.15 ± 0.10	14.1 ± 0.04 ^bcd^	0.9918 ± 0.08	0.1 ± 0.01 ^bcd^	123 ± 0.10 ^g^	41 ± 0.20 ^j^
V36	7.49 ± 0.00 ^abc^	0.33 ± 0.05	3.16 ± 0.20	14.6 ± 0.01 ^ab^	0.9914 ± 0.02	0.1 ± 0.00 ^bcd^	120 ± 0.02 ^i^	40 ± 0.06 ^j^
Analysis of variance								
*p*-value	<0.0001	0.041	0.842	<0.001	1.000	<0.0001	<0.0001	<0.0001
Robust test of equality of means: Welch statistic								
*p*-value	<0.0001	<0.0001	0.268	<0.0001	1.000	<0.0001	<0.0001	<0.0001
Brown-Forsythe								
*p*-value	<0.0001	<0.0001	0.797	<0.0001	1.000	<0.0001	<0.0001	<0.0001
Effect size measures								
ŋp^2^	0.752	0.439	0.265	0.912	0.000	0.965	0.999	0.998

TA—total acidity; VA—volatile acidity; AS—alcoholic strength; D—density; RS—reducing sugars; SO_2_—sulphur dioxide. Values are expressed as mean ± standard deviation of three analytical replicates. Differences among samples were assessed by one-way ANOVA, followed by Tukey’s Honest Significant Difference (HSD) test when significant effects were observed (*p* < 0.05). Means sharing at least one common letter are not significantly different at *p* < 0.05.

**Table 3:** Concentrations of phenolic compounds in Fetească regală samples.

Sample	C1	C2	C3	C4	C5	C6	C7	C8	C9	C10	C11	C12	C13	C14	C15	C16
V0	0.67 ± 0.10	0.35 ± 0.08	0.01 ± 0.01 ^g^	1.54 ± 0.03 ^r^	0.49 ± 0.00	0.32 ± 0.01	0.06 ± 0.02	0.07 ± 0.05	0.02 ± 0.00	nd	nd	36.31 ± 0.10 ^o^	1.13 ± 0.24	0.22 ± 0.07	0.08 ± 0.10	0.24 ± 0.45
V1	0.53 ± 0.12	0.29 ± 0.00	0.02 ± 0.00 ^defg^	2.47 ± 0.08 ^lmno^	0.62 ± 0.08	0.20 ± 0.09	0.05 ± 0.04	0.04 ± 0.03	0.01 ± 0.00	0.01 ± 0.06	0.40 ± 0.04 ^efg^	37.34 ± 0.23 ^i^	1.33 ± 0.12	0.19 ± 0.02	0.13 ± 0.07	0.25 ± 0.08
V2	0.59 ± 0.4	0.28 ± 0.02	0.02 ± 0.02 ^efg^	3.45 ± 0.05 ^cde^	0.60 ± 0.05	0.21 ± 0.01	0.05 ± 0.02	0.05 ± 0.00	0.03 ± 0.10	nd	nd	36.86 ± 0.20 ^jkl^	1.11 ± 0.10	0.21 ± 0.10	0.14 ± 0.09	0.21 ± 0.12
V3	0.47 ± 0.15	0.26 ± 0.00	0.07 ± 0.04 ^bcdef^	2.40 ± 0.00 ^mno^	0.58 ± 0.02	0.13 ± 0.08	0.03 ± 0.01	0.03 ± 0.20	0.04 ± 0.20	0.01 ± 0.00	0.84 ± 0.10 ^bc^	36.71 ± 0.11 ^klmn^	1.20 ± 0.45	0.19 ± 0.05	0.12 ± 0.02	0.17 ± 0.00
V4	0.58 ± 0.00	0.32 ± 0.04	0.03 ± 0.00 ^defg^	2.23 ± 0.15 ^opq^	0.60 ± 0.01	0.22 ± 0.00	0.05 ± 0.12	0.04 ± 0.01	0.03 ± 0.02	0.01 ± 0.00	0.14 ± 0.11 ^hij^	37.42 ± 0.13 ^hi^	1.18 ± 0.12	0.15 ± 0.05	0.13 ± 0.03	0.21 ± 0.01
V5	0.62 ± 0.02	0.32 ± 0.00	0.04 ± 0.05 ^defg^	2.48 ± 0.01 ^lmno^	0.65 ± 0.05	0.26 ± 0.02	0.03 ± 0.25	0.04 ± 0.00	0.02 ± 0.01	0.01 ± 0.01	nd	36.98 ± 0.22 ^j^	1.20 ± 0.09	0.24 ± 0.15	0.13 ± 0.02	0.26 ± 0.05
V6	0.61 ± 0.15	0.30 ± 0.20	0.06 ± 0.15 ^bcdefg^	2.23 ± 0.08 ^opq^	0.65 ± 0.01	0.17 ± 0.00	0.02 ± 0.01	0.05 ± 0.03	0.03 ± 0.01	0.01 ± 0.00	0.56 ± 0.04 ^de^	36.90 ± 0.14 ^jk^	1.24 ± 0.16	0.17 ± 0.03	0.15 ± 0.01	0.26 ± 0.04
V7	0.51 ± 0.08	0.28 ± 0.10	0.06 ± 0.01 ^bcdefg^	3.37 ± 0.12 ^def^	0.62 ± 0.10	0.16 ± 0.04	0.02 ± 0.02	0.03 ± 0.01	0.02 ± 0.10	0.01 ± 0.15	0.66 ± 0.12 ^cd^	36.62 ± 0.22 ^lmn^	1.13 ± 0.50	0.15 ± 0.06	0.09 ± 0.20	0.21 ± 0.07
V8	0.62 ± 0.12	0.35 ± 0.02	0.03 ± 0.00 ^defg^	4.00 ± 0.45 ^b^	0.58 ± 0.04	0.24 ± 0.00	0.04 ± 0.00	0.04 ± 0.02	0.03 ± 0.01	0.01 ± 0.30	0.11 ± 0.04 ^ij^	37.38 ± 0.21 ^i^	1.18 ± 0.30	0.20 ± 0.07	0.11 ± 0.03	0.24 ± 0.06
V9	0.44 ± 0.00	0.23 ± 0.05	0.10 ± 0.02 ^ab^	2.88 ± 0.20 ^ij^	0.61 ± 0.06	0.11 ± 0.20	0.03 ± 0.02	0.04 ± 0.01	0.02 ± 0.00	0.01 ± 0.08	1.44 ± 0.01 ^a^	36.98 ± 0.22 ^j^	1.22 ± 0.00	0.14 ± 0.08	0.12 ± 0.08	0.29 ± 0.40
V10	0.53 ± 0.01	0.30 ± 0.01	0.02 ± 0.00 ^defg^	3.20 ± 0.30 ^efgh^	0.56 ± 0.03	0.20 ± 0.10	0.04 ± 0.01	0.04 ± 0.02	0.03 ± 0.01	0.01 ± 0.12	0.23 ± 0.11 ^ghij^	36.98 ± 0.22 ^j^	1.13 ± 0.15	0.16 ± 0.09	0.13 ± 0.20	0.18 ± 0.00
V11	0.51 ± 0.05	0.31 ± 0.01	0.02 ± 0.02 ^efg^	3.59 ± 0.40 ^cd^	0.62 ± 0.20	0.16 ± 0.02	0.04 ± 0.04	0.04 ± 0.00	0.02 ± 0.00	0.01 ± 0.01	0.09 ± 0.12 ^ij^	37.46 ± 0.14 ^ghi^	1.24 ± 0.30	0.19 ± 0.10	0.12 ± 0.03	0.25 ± 0.07
V12	0.51 ± 0.04	0.27 ± 0.02	0.10 ± 0.00 ^ab^	2.88 ± 0.05 ^ij^	0.60 ± 0.10	0.12 ± 0.05 ^c^	0.03 ± 0.00	0.03 ± 0.01	0.02 ± 0.00	nd	1.30 ± 0.03 ^a^	37.85 ± 0.14 ^bcde^	1.24 ± 0.08	0.10 ± 0.04	0.10 ± 0.03	0.19 ± 0.08
V13	0.52 ± 0.20	0.27 ± 0.00	0.03 ± 0.05 ^defg^	2.82 ± 0.09 ^ijk^	0.68 ± 0.00	0.17 ± 0.01	0.03 ± 0.10	0.04 ± 0.05	0.01 ± 0.01	nd	0.35 ± 0.00 ^efgh^	37.69 ± 0.19 ^defg^	1.20 ± 0.40	0.16 ± 0.08	0.09 ± 0.08	0.25 ± 0.02
V14	0.55 ± 0.05	0.30 ± 0.03	0.03 ± 0.04 ^defg^	3.41 ± 0.10 ^def^	0.62 ± 0.12	0.20 ± 0.01	0.05 ± 0.01	0.04 ± 0.00	0.03 ± 0.01	0.01 ± 0.02	0.08 ± 0.02 ^ij^	38.01 ± 0.16 ^b^	1.37 ± 0.34	0.22 ± 0.09	0.14 ± 0.05	0.18 ± 0.03
V15	0.50 ± 0.15	0.26 ± 0.10	0.06 ± 0.00 ^bcdefg^	2.34 ± 0.02 ^nop^	0.63 ± 0.07	0.14 ± 0.20	0.04 ± 0.01	0.03 ± 0.01	0.02 ± 0.01	0.01 ± 0.12	0.72 ± 0.01 ^cd^	37.85 ± 0,18 ^bcde^	1.29 ± 0.40	0.16 ± 0.30	0.15 ± 0.06	0.23 ± 0.02
V16	0.64 ± 0.10	0.31 ± 0.08	0.03 ± 0.10 ^defg^	2.39 ± 0.05 ^mno^	0.60 ± 0.02	0.22 ± 0.03	0.06 ± 0.00	0.05 ± 0.03	0.01 ± 0.00	0.01 ± 0.35	0.14 ± 0.01 ^hij^	37.30 ± 0.17 ^i^	1.29 ± 0.43	0.24 ± 0.04	0.19 ± 0.08	0.17 ± 0.00
V17	0.61 ± 0.20	0.32 ± 0.00	0.02 ± 0.01 ^efg^	2.82 ± 0.10 ^ijk^	0.63 ± 0.20	0.20 ± 0.08	0.03 ± 0.00	0.03 ± 0.04	0.02 ± 0.02	0.01 ± 0.15	nd	36.78 ± 0.21 ^jklm^	1.20 ± 0.02	0.19 ± 0.05	0.08 ± 0.20	0.15 ± 0.01
V18	0.57 ± 0.00	0.27 ± 0.01	0.04 ± 0.03 ^cdefg^	2.30 ± 0.08 ^nopq^	0.62 ± 0.00	0.18 ± 0.20	0.04 ± 0.02	0.04 ± 0.00	0.02 ± 0.00	0.01 ± 0.10	0.72 ± 0.03 ^cd^	36.47 ± 0.19 ^no^	1.31 ± 0.05	0.21 ± 0.09	0.09 ± 0.03	0.21 ± 0.05
V19	0.50 ± 0.10	0.27 ± 0.02	0.08 ± 0.02 ^abcd^	3.71 ± 0.00 ^c^	0.59 ± 0.30	0.15 ± 0.03	0.03 ± 0.00	0.04 ± 0.01	0.01 ± 0.00	0.0 ± 0.081	0.73 ± 0.01 ^cd^	37.69 ± 0.24 ^defg^	1.18 ± 0.10	0.20 ± 0.10	0.14 ± 0.03	0.24 ± 0.00
V20	0.6 ± 0.01	0.32 ± 0.20	0.02 ± 0.00 ^efg^	4.41 ± 0.01 ^a^	0.70 ± 0.30	0.22 ± 0.03	0.03 ± 0.01	0.04 ± 0.01	0.03 ± 0.00	0.01 ± 0.15	0.09 ± 0.03 ^ij^	38.92 ± 0.15 ^a^	1.22 ± 0.08	0.20 ± 0.02	0.15 ± 0.00	0.34 ± 0.01
V21	0.49 ± 0.01	0.25 ± 0.00	0.13 ± 0.02 ^a^	3.01 ± 0.02 ^hi^	0.64 ± 0.03	0.14 ± 0.08	0.04 ± 0.01	0.03 ± 0.01	0.02 ± 0.02	0.01 ± 0.05	1.39 ± 0.08 ^a^	37.50 ± 0.21 ^fghi^	1.33 ± 0.00	0.20 ± 0.05	0.12 ± 0.04	0.16 ± 0.03
V22	0.55 ± 0.08	0.29 ± 0.02	0.04 ± 0.00 ^defg^	3.39 ± 0.40 ^def^	0.67 ± 0.04	0.18 ± 0.05	0.06 ± 0.03	0.04 ± 0.04	0.03 ± 0.00	0.01 ± 0.02	0.28 ± 0.04 ^ghi^	37.38 ± 0.12 ^i^	1.20 ± 0.01	0.22 ± 0.10	0.11 ± 0.00	0.23 ± 0.04
V23	0.60 ± 0.04	0.30 ± 0.03	0.02 ± 0.04 ^efg^	4.08 ± 0.05 ^b^	0.65 ± 0.02	0.20 ± 0.00	0.04 ± 0.08	0.04 ± 0.02	0.03 ± 0.04	0.01 ± 0.15	0.10 ± 0.03 ^ij^	37.29 ± 0.24 ^i^	1.21 ± 0.80	0.20 ± 0.50	0.12 ± 0.20	0.23 ± 0.01
V24	0.45 ± 0.03	0.25 ± 0.01	0.10 ± 0.00 ^ab^	3.06 ± 0.09 ^ghi^	0.62 ± 0.30	0.08 ± 0.04	0.02 ± 0.04	0.03 ± 0.00	0.02 ± 0.00	nd	1.30 ± 0.80 ^a^	37.65 ± 0.22 ^efgh^	1.26 ± 0.70	0.15 ± 0.06	0.14 ± 0.10	0.23 ± 0.08
V00	0.64 ± 0.04	0.34 ± 0.00	0.01 ± 0.20 ^g^	1.57 ± 0.10 ^r^	0.61 ± 0.01	0.28 ± 0.00	0.07 ± 0.20	0.05 ± 0.01	0.03 ± 0.20	nd	nd	36.55 ± 0.20 ^mno^	1.20 ± 0.01	0.18 ± 0.01	0.13 ± 0.02	0.23 ± 0.04
V25	0.53 ± 0.02	0.30 ± 0.00	0.03 ± 0.10 ^defg^	2.37 ± 0.02 ^mnop^	0.64 ± 0.05	0.19 ± 0.08	0.03 ± 0.00	0.04 ± 0.80	0.02 ± 0.10	0.01 ± 0.05	0.29 ± 0.02 ^fghi^	37.46 ± 0.13 ^ghi^	1.24 ± 0.03	0.18 ± 0.05	0.13 ± 0.05	0.23 ± 0.03
V26	0.65 ± 0.01	0.34 ± 0.20	0.03 ± 0.20 ^defg^	2.71 ± 0.05 ^jkl^	0.67 ± 0.04	0.250.04	0.06 ± 0.01	0.05 ± 0.70	0.02 ± 0.02	0.01 ± 0.02	nd	37.97 ± 0.26 ^bc^	1.24 ± 0.08	0.24 ± 0.04	0.15 ± 0.90	0.25 ± 0.04
V27	0.59 ± 0.02	0.29 ± 0.02	0.07 ± 0.05 ^abcde^	2.11 ± 0.10 ^pq^	0.62 ± 0.01	0.16 ± 0.03	0.04 ± 0.04	0.05 ± 0.08	0.02 ± 0.05	nd	0.65 ± 0.10 ^cd^	36.90 ± 0.15 ^jk^	1.20 ± 0.04	0.18 ± 0.05	0.10 ± 0.30	0.24 ± 0.02
V28	0.59 ± 0.01	0.31 ± 0.03	0.01 ± 0.01 ^g^	2.05 ± 0.15 ^q^	0.62 ± 0.05	0.21 ± 0.04	0.05 ± 0.02	0.04 ± 0.04	0.03 ± 0.01	0.01 ± 0.04	0.10 ± 0.15 ^ij^	37.34 ± 0.21 ^i^	1.35 ± 0.04	0.15 ± 0.03	0.10 ± 0.02	0.22 ± 0.01
V29	0.56 ± 0.05	0.34 ± 0.01	0.03 ± 0.01 ^defg^	2.45 ± 0.04 ^lmno^	0.65 ± 00.00	0.19 ± 0.03	0.03 ± 0.01	0.03 ± 0.03	0.03 ± 0.00	nd	nd	37.54 ± 0.17 ^fghi^	1.13 ± 0.01	0.21 ± 0.00	0.12 ± 0.04	0.19 ± 0.02
V30	0.57 ± 0.04	0.30 ± 0.00	0.04 ± 0.02 ^cdefg^	2.31 ± 0.08 ^nopq^	0.61 ± 0.02	0.15 ± 0.04	0.03 ± 0.08	0.03 ± 0.02	0.03 ± 0.02	nd	0.41 ± 0.08 ^efg^	37.73 ± 0.09 ^cdef^	1.34 ± 0.08	0.24 ± 0.20	0.10 ± 0.08	0.24 ± 0.01
V31	0.56 ± 0.20	0.30 ± 0.03	0.05 ± 0.00 ^bcdefg^	3.17 ± 0.05 ^fgh^	0.64 ± 0.03	0.21 ± 0.02	0.03 ± 0.04	0.05 ± 0.01	0.02 ± 0.03	0.01 ± 0.04	0.51 ± 0.03 ^def^	37.30 ± 0.17 ^i^	1.24 ± 0.04	0.18 ± 0.01	0.12 ± 0.03	0.24 ± 0.05
V32	0.56 ± 0.00	0.29 ± 0.04	0.03 ± 0.00 ^defg^	3.28 ± 0.04 ^efg^	0.67 ± 0.01	0.17 ± 0.01	0.04 ± 0.03	0.02 ± 0.05	0.03 ± 0.00	0.01 ± 0.20	0.08 ± 0.08 ^ij^	37.41 ± 0.19 ^hi^	1.20 ± 0.03	0.22 ± 0.00	0.08 ± 0.08	0.18 ± 0.05
V33	0.46 ± 0.02	0.25 ± 0.04	0.10 ± 0.10 ^abc^	2.80 ± 0.20 ^ijk^	0.66 ± 0.00	0.12 ± 0.02	0.03 ± 0.04	0.03 ± 0.00	0.03 ± 0.00	nd	1.07 ± 0.04 ^b^	37.93 ± 0.24 ^bcd^	1.20 ± 0.04	0.18 ± 0.03	0.08 ± 0.04	0.27 ± 0.15
V34	0.55 ± 0.01	0.31 ± 0.01	0.03 ± 0.01 ^defg^	2.63 ± 0.05 ^jklm^	0.67 ± 0.03	0.17 ± 0.50	0.03 ± 0.02	0.03 ± 0.02	0.02 ± 0.01	0.01 ± 0.15	0.19 ± 0.05 ^ghij^	37.73 ± 0.11 ^cdef^	1.16 ± 0.00	0.16 ± 0.01	0.11 ± 0.05	0.23 ± 0.10
V35	0.59 ± 0.01	0.32 ± 0.02	0.02 ± 0.05 ^fg^	2.55 ± 0.15 ^klmn^	0.67 ± 0.04	0.22 ± 0.00	0.05 ± 0.01	0.05	0.02 ± 0.00	nd	0.00 ± 0.04 ^j^	36.69 ± 0.08 ^defg^	1.18 ± 0.20	0.17 ± 0.02	0.12 ± 0.60	0.24 ± 0.05
V36	0.49 ± 0.0	0.27 ± 0.00	0.06 ± 0.04 ^bcdefg^	2.37 ± 0.40 ^mnop^	0.63 ± 0.03	0.13 ± 0.03	0.03 ± 0.02	0.03 ± 0.0	0.01 ± 0.01	0.01 ± 0.00	0.69 ± 0.02 ^cd^	37.34 ± 0.21 ^i^	1.22 ± 0.01	0.17 ± 0.01	0.13 ± 0.80	0.23 ± 0.80
Analysis of variance																
*p*-values	0.126	0.955	0.001	<0.0001	0.999	0.827	0.281	0.978	0.715	1.000	<0.0001	<0.0001	1.000	1.000	1.000	0.092
Robust test of equality of means Welch statistic																
*p*-values	<0.0001	0.002	0.001	<0.0001	0.003	<0.0001	0.406	0.975	0.966	1.000	<0.0001	<0.0001	0.001	0.863	0.171	<0.0001
Brown-Forsythe																
*p*-values	0.223	0.879	0.124	<0.0001	0.986	0.721	0.335	0.951	0.669	1.000	0.011	<0.0001	1.000	1.000	0.999	0.178
Effect size measure																
ŋp^2^	0.400	0.523	0.958	0.161	0.269	0.362	0.210	0.291	0.001	0.933	0.942	0.083	0.075	0.063	0.411	0.400

Values are expressed as mean ± standard deviation of three analytical replicates. Differences among means were assessed using one-way ANOVA followed by Tukey’s Honest Significant Difference (HSD) test (*p* < 0.05). Means sharing at least one common letter are not significantly different. Not detected (nd) values were excluded from statistical analysis. All values are expressed in mg L^−1^. Compounds were identified as follows: C1—epicatechin; C2—catechin; C3—syringic acid; C4—gallic acid; C5—protocatechuic acid; C6—procyanidin B1; C7—procyanidin B2; C8—procyanidin B3; C9—procyanidin B4; C10—esculetin; C11—syringaldehyde; C12—caftaric acid; C13—caffeic acid; C14—*p*-coumaric acid; C15—*trans*-resveratrol; C16—*cis*-resveratrol. Robust tests (Welch and Brown–Forsythe) were also considered due to deviations from normality.

**Table 4:** Comparative economic cost of the different maturation variants (€ L^−1^).

Sample Code	Treatment	Time	Dose (g L^−1^)	Cost * (€ L^−1^)
V0	Control (no oak)	-	-	0.05 ± 0.01 ^a^
V1–V3	Granules (fresh–medium)	10 days	1	0.21 ± 0.10 ^b^
V4–V6	Chips (fresh–medium)	10 days	1	0.23 ± 0.11 ^b^
V7–V9	Granules (fresh–medium)	20 days	1	0.41 ± 0.20 ^c^
V10–V12	Chips (fresh–medium)	20 days	1	0.45 ± 0.22 ^c^
V13–V15	Granules (fresh–medium)	10 days	2	0.24 ± 0.11 ^b^
V16–V18	Chips (fresh–medium)	10 days	2	0.26 ± 0.12 ^b^
V19–V21	Granules (fresh–medium)	20 days	2	0.52 ± 0.25 ^d^
V22–V24	Chips (fresh–medium)	20 days	2	0.56 ± 0.27 ^d^
V00	Ultrasound control	15 min	-	0.06 ± 0.02 ^a^
V25–V27	Granules + ultrasound	~15 min	1	0.05 ± 0.02 ^a^
V28–V30	Chips + ultrasound	~15 min	1	0.06 ± 0.02 ^a^
V31–V33	Granules + ultrasound	~15 min	2	0.06 ± 0.02 ^a^
V34–V36	Chips + ultrasound	~15 min	2	0.07 ± 0.02 ^a^
-	Barrel ageing (literature)	6–12 months	-	4.25 ± 1.50 ^e^

* Values represent mean ± standard deviation (€ L^−1^). Different letters indicate statistically significant differences among samples (*p* < 0.05). Values are expressed as mean ± standard deviation of three replicates. Samples sharing at least one common letter are not significantly different from each other according to Tukey’s Honest Significant Difference (HSD) test. Time cost was estimated at 0.01–0.03 €/L/day; ultrasound treatments assume a negligible time cost.

**Table 5:** Cost per unit of gallic acid (€ mg^−1^) across maturation variants.

Variant	Cost (€ L^−1^)	Gallic Acid (mg L^−1^)	Cost Efficiency (€ mg^−1^)
V0	0.05	1.54	0.032 ^b^
V1	0.21	2.47	0.085 ^c^
V2	0.21	3.45	0.061 ^b^
V3	0.21	2.4	0.087 ^c^
V4	0.23	2.23	0.103 ^c^
V5	0.23	2.48	0.093 ^c^
V6	0.23	2.23	0.103 ^c^
V7	0.41	3.37	0.122 ^c^
V8	0.41	4.0	0.102 ^c^
V9	0.41	2.88	0.142 ^c^
V10	0.45	3.2	0.141 ^c^
V11	0.45	3.59	0.125 ^c^
V12	0.45	2.88	0.156 ^d^
V13	0.24	2.82	0.085 ^c^
V14	0.24	3.41	0.070 ^c^
V15	0.24	2.34	0.103 ^c^
V16	0.26	2.39	0.109 ^c^
V17	0.26	2.82	0.092 ^c^
V18	0.26	2.3	0.113 ^c^
V19	0.52	3.71	0.140 ^c^
V20	0.52	4.41	0.118 ^c^
V21	0.52	3.01	0.173 ^d^
V22	0.56	3.39	0.165 ^d^
V23	0.56	4.08	0.137 ^c^
V24	0.56	3.06	0.183 ^d^
V25	0.05	2.37	0.021 ^a^
V26	0.05	2.71	0.018 ^a^
V27	0.05	2.11	0.024 ^a^
V28	0.06	2.05	0.029 ^a^
V29	0.06	2.45	0.024 ^a^
V30	0.06	2.31	0.026 ^a^
V31	0.06	3.17	0.019 ^a^
V32	0.06	3.28	0.018 ^a^
V33	0.06	2.8	0.021 ^a^
V34	0.07	2.63	0.027 ^a^
V35	0.07	2.55	0.027 ^a^
V36	0.07	2.37	0.03 ^a^

Cost-efficiency calculated as €/L divided by gallic acid concentration (mg L^−1^). Lower values indicate higher economic efficiency. Different letters indicate statistically significant differences among samples (*p* < 0.05).

## Data Availability

The original contributions presented in the study are included in the article, further inquiries can be directed to the corresponding authors.
